# Posttraumatic Stress Disorder and Hypertension in Vietnam Veteran Men and Women

**DOI:** 10.1093/geroni/igaf122.626

**Published:** 2025-12-31

**Authors:** Avron Spiro, Kelsey Serier, Hannah Burns, Rachel Kimerling, Anica Pless Kaiser, Kathryn Magruder, Brian Smith

**Affiliations:** Boston University - Chobanian & Avedisian School of Medicine, Waban, United States; VA Boston Health Care System, Boston, Massachusetts, United States; VA Boston Health Care System, Boston, Massachusetts, United States; VA Palo Alto Health Care System, Palo Alto, California, United States; VA Boston Health Care System, Boston, Massachusetts, United States; Medical University of South Carolina, Charleston, South Carolina, United States; National Center for PTSD Women’s Health Sciences Division, Boston, Massachusetts, United States

## Abstract

Hypertension is a risk factor for cardiovascular disease, a leading cause of death for older adults. Posttraumatic stress disorder (PTSD) may increase the likelihood of developing hypertension. However, this association has seldom been studied in older adults and infrequently among veterans. The present study used data from two cohorts of older adult Vietnam era veterans: over 4104 women (Mage= 67 years) and 5767 men Mage= 62 years). Respondents completed by telephone a structured clinical interview assessing lifetime PTSD. Information on self-reported hypertension diagnosis, age of onset, and past-year treatment, as well as on covariates was obtained by mail survey. Weighted logistic regression analyses adjusted for relevant covariates revealed an association between PTSD and higher likelihood of hypertension in male veterans (OR = 1.57; 95% CI [1.30, 1.91]). There was no association between lifetime PTSD and hypertension in female veterans (OR = 0.93; 95% CI [0.78, 1.11]). Exploratory secondary analyses suggested an association between PTSD and hypertension onset in early and middle adulthood only in men. PTSD was not associated with past-year hypertension treatment for women or men. Overall, these findings suggest that PTSD may contribute to hypertension risk in older male veterans, but no contribution was found for female veterans. Prospective research is needed to confirm these findings and further clarify the relationship between PTSD and hypertension to inform veteran clinical care.

